# The Influence of Titanium Dioxide (TiO_2_) Particle Size and Crystalline Form on the Microstructure and UV Protection Factor of Polyester Substrates

**DOI:** 10.3390/polym16040475

**Published:** 2024-02-08

**Authors:** María Cot, Gabriela Mijas, Remedios Prieto-Fuentes, Marta Riba-Moliner, Diana Cayuela

**Affiliations:** 1 Terrassa Institute of Textile Research and Industrial Cooperation (INTEXTER), Universitat Politècnica de Catalunya (UPC), 08222 Terrassa, Spain; 2 Department of Materials Science and Engineering (CEM), Universitat Politècnica de Catalunya (UPC), 08222 Terrassa, Spain; 3 Fundación Asociación de Becarios Retornados EC (ABREC), Quito 170518, Ecuador

**Keywords:** ultraviolet protection factor (UPF), titanium dioxide (TiO_2_), anatase, rutile, polyethylene terephthalate (PET), POY multifilament, differential scanning calorimeter (DSC), crystallinity, knitted fabric

## Abstract

The inclusion of particles in a polymeric substrate to achieve certain properties is a well-known practice. In the case of textile substrates, this practice may deeply affect the structure of the produced yarns, as even a filament with no textile applications can be obtained. In this manuscript, titanium dioxide (TiO_2_) particles were incorporated into polyester (PET) chips and the influence of these fillers on the properties of yarn and fabric, and the ultraviolet protection factor (UPF) was assessed. For this purpose, rutile and anatase crystalline forms of TiO_2_, as well as the size of the particles, were evaluated. Moreover, parameters such as mechanical properties, orientation of the macromolecules and thermal behavior were analyzed to ensure that the textile grade is maintained throughout the production process. The results showed that the inclusion of micro- and nanoparticles of TiO_2_ decreases the molecular weight and tenacity of PET. Also, although orientation and crystallinity varied during the textile process, the resulting heatset fabrics did not present important differences in those parameters. Finally, the attainment of textile-grade PET-TiO_2_ fabrics with UPF indexes of 50+ with both rutile and anatase and micro- and nano-sized TiO_2_ forms was demonstrated.

## 1. Introduction

In recent years, there has been a significant surge in the application of nanotechnology within the textile industry, particularly in the realm of incorporating substances to enhance functionality [1]. For example, silver nanoparticles have been used to provide antibacterial properties [2], titanium dioxide (TiO_2_) to block UV rays [3], and zinc oxide (ZnO) for self-cleaning [4]. The conventional methods used to apply these substances have been surface treatments, which provide fabrics with different properties in a satisfactory mode but, occasionally, they do not have permanent effects, and the substrates lose their function after washing or a certain usage time. In most cases, nanoparticles have been added as a finishing process in the textile chain, although attempts have been made to incorporate them in earlier stages, such as spinning, using the twisting of fibers in the yarn to retain the nanoparticles inside. Various publications have discussed the application of nanoparticles by depositing them on the surface of fibers to modify some of their properties [5,6,7,8,9]. The inclusion of fillers during the production of polyethylene terephthalate (PET) yarn by a melt spinning process improves the quality of the composite, with the possibility of providing more permanent multifunctional properties to textile materials. The size of those particles is a crucial parameter that determines the final properties of the system. As the size of the particles decreases, there is a greater tendency for agglomeration and, simultaneously, an increase in specific surface area, leading to thermodynamic instability [10]. To achieve uniform dispersions and avoid aggregation, it is essential to regulate these parameters when incorporating particles into a polymeric matrix. The use of dispersing agents is crucial in ensuring homogeneity throughout the process [11,12].

Most of the applications discussed in the literature focus on the utilization of ceramic nanoparticles in polymeric matrices, typically in the form of films or substrates produced through injection or molding processes. The production of filament-shaped composites, which exhibit unique structures and properties, is limited [4,13,14,15]. Nevertheless, the progress in functional fiber development plays a pivotal role in the advancement of textiles for technical applications.

Titanium dioxide (TiO_2_) is an inorganic, non-toxic compound, chemically stable against exposure to both elevated temperatures and UV rays, abundant, and relatively cheap [16,17]. Additionally, TiO_2_ can be obtained in various crystalline forms, such as rutile, anatase and brookite, with the first two being the most widely used at the industrial level. Moreover, the catalytic activity of TiO_2_ is greatly enhanced when the particle size is lower than 20 nm. Unfortunately, few synthesis techniques allow for the production of such small sizes (mainly particles < 10 nm) in a reproducible way, limiting the range of the particles currently studied [17,18].

Previous studies have primarily focused on exploring the dispersion, thermal properties, mechanical properties, ultraviolet protection, antibacterial properties, and other relevant factors associated with the inclusion of TiO_2_ particles during the melt spinning of PET [13,14,15]. However, it is important to evaluate the influence of particle size and polymorphism on the aforementioned properties. On an industrial scale, the results of this study are relevant because TiO_2_ is used as a matting agent in the synthesis of plastics and textile fibers [16].

In this study, TiO_2_ was applied to textile fibers to produce PET fabrics that block UV rays, thus obtaining radiation-protective textiles across a wide range of wavelengths. For this, TiO_2_ was incorporated during PET spinning and the crystalline forms of rutile and anatase were considered to study the effect of polymorphism, followed by the evaluation of size effect using micro- and nano-sized particles. The mechanical properties, orientation, crystallinity, and thermal behavior of the yarns produced were evaluated. Finally, knitted fabrics were produced and the UPF and UPF index were analyzed.

## 2. Experimental Section

### 2.1. Materials

Titanium dioxide (TiO_2_) particles, later incorporated in the spinning of POY PET yarns, were rutile and anatase crystalline forms in micro- and nanometric sizes supplied by Zeus Química, S.A. (Barcelona, Spain). The characteristics of the particles, as provided by the manufacturers [19,20,21,22], are shown in Table 1 and TEM images are available in a previous work [23].

The dispersing agent used was LICOWAX E (Clariant)( (Sant Joan Despí, Spain), an ester of montanic acids with multifunctional alcohols (MAWMA), which was found to be suitable for dispersing TiO_2_ particles in a PET matrix in a previous work (Table 2) [24,25].

Partially oriented yarns (POY) of PET multifilament (48 filaments) containing TiO_2_ particles were manufactured by IQAP Masterbatch Group S.L. (Masies de Roda, Spain) by melt spinning using an extrusion temperature of 295 °C, a pressure of 80 bar, and a winder collection speed of 3000 m/min. The composition of these POY including TiO_2_ particles is presented in Table 3. In a previous study, a discontinuity in the extrusion outflow of some mixtures with concentrations of 3% TiO_2_ was found to be caused by a decrease in viscosity, which prevented the spinning of the yarns. Consequently, the preparation of the yarns had a maximum concentration limit of 2% TiO_2_ [26]. It should be noted that the concentration of TiO_2_ nanoparticles in the yarn containing RN particles is lower compared to that of the other yarns. This is due to the fact that these particles caused a decrease in the viscosity of the PET matrix. Consequently, the quantity of nanoparticles had to be slightly reduced in order to maintain the appropriate flow of polymer and ensure the continuity required for yarn production.

### 2.2. Treatments

After spinning, the yarns were drawn with a stretch ratio of 1:2 at 190 °C. This post-spinning stretching was applied to orientate the filaments in the direction of the fiber axis and to give a proper crystallinity, making the yarns suitable for use as textile fibers. Afterwards, plain knitted fabrics were produced using a circular knitting machine (90 mm and 167 needles). The averaged characteristics of the produced fabrics were as follows: stitch length 3.23 mm/stitch; thickness 2.24 ± 0.011 mm, 16.1 ± 1.0 courses/cm, 10.8 ± 0.58 wales/cm, and 0.89 ± 0.01 g/m^2^.

Finally, the fabrics were heatset to thermally stabilize the substrate. The process was performed in a stenter Ernst Benz AG (Zürich, Switzerland). The heatsetting conditions were a temperature of 190 °C for 1 min without tension.

### 2.3. Characterization

Scanning Electron Microscopy (SEM)

Micrographs of the yarns were obtained with a Phenom World (Eindhoven, The Netherlands) 800-03103-02 scanning electron microscope. Samples were previously Au/Pd coated.

Molecular weight determination

The determination of the molecular weight was performed by gel permeation chromatography (GPC) on a Perkin Elmer (Norwalk, CN, USA) 200 High Performance Liquid Chromatograph equipped with an Agilent PLgel 5 µm Mixed-D column 300 × 7.5 mm preceded by an Agilent (Stockport, UK) Oligopore column 6 µm 300 × 7.5 mm. The system was calibrated with Waters (Milford, CN, USA) polystyrene standards, with an average molecular weight in the range of 2350–200,000 g/mol. Then, 3 mg of washed yarn were placed in a test tube with 0.2 mL of *o*-chlorophenol (>99%, Sigma-Aldrich, Steinheim, Germany). After dissolving the yarn and cooling the tubes, 1.8 mL of chloroform (HPLC grade, stabilized with amylene, approx. 150 ppm, Scharlau, Barcelona, Spain) was added and the mixture was stirred. The resulting solution was then filtered (PTFE filter, 0.45 μm) and transferred to a vial. Analyses were conducted at 40 °C with chloroform (HPLC grade, Scharlau, Barcelona, Spain) as the mobile phase at a flow rate of 1 mL/min. The TotalChrom Navigator–TurboSEC v. 6.3.1.0504 software (Perkin Elmer, Norwalk, CN, USA) was used to calculate the molecular weight in number, the molecular weight in weight, and polydispersity.

Yarns and fabric parameters

Previous to characterization, the samples were conditioned for 48 h at 20 ± 2 °C and relative humidity 65 ± 4% according to the ISO 139:2005 standard [27].

The linear density of the yarns was determined according to the ISO 2060:1996 standard [28], the surface mass was obtained according to the UNE-EN 12127:1998 standard [29], and the thickness was calculated according to the ISO 5084:1997 standard [30] using a pressure of 100 g/cm^2^ in an Adamel Lhomargy Creusot-Loire (Le Creusot, France) compression meter.

Dynamometric tests were carried out in a Uster (Uster, Switzerland) TensoKid automatic PE 4056 equipment, following the ISO 2062:2010 standard [31] with a gauge length of 250 mm, a test speed of 250 mm/min, pretension of 0.5 CN/tex, a breaking strength of 80%, and 50 samples per yarn.

Determination of the orientation of the molecules (sonic modulus)

Sonic modulus determines the molecular orientation along the fiber axis. By measuring the speed of sound traveling longitudinally through the fiber, and, later, by calculating the sound modulus before and after drawing the yarns, the increase in molecular orientation along the fiber axis was determined [32]. A HM Morgan Co., Inc. (Cambridge, MA, USA) Dynamic Modulus Tester PPM–5R was used for that purpose.

Differential Scanning Calorimetry (DSC)

The DSC technique was employed to determine the crystallinity of the samples. A Perkin Elmer (Norwalk, CN, USA) DSC7/TAC7 calorimeter was used, and the conditions of the experiment were as follows: holding at 40 °C for 5 min, first heating from 40 °C to 300 °C at 20 °C/min, holding at 300 °C for 5 min, and cooling from 300 °C to 40 °C at 20 °C/min. Nitrogen (2 kg/cm^2^) was used as the purge gas.

In the DSC steps, the first heating was used to evaluate the microstructure of the non-drawn and drawn yarns and of the heatset fabrics, and to determine the crystallinity from the melting enthalpies of the samples. This first heating was also used to eliminate the thermal history of the polymer and, so, the study of the crystallization process, which solely depended on the blend and not the structure, was conducted by obtaining cooling thermograms in all instances. To calculate the total melting enthalpies ($∆H_{T})$ and avoid baseline errors, the methodology detailed by Cayuela et al. [33] was applied. A pure PET thermogram was subtracted from the thermogram obtained from each of the samples containing particles and the resulting thermogram was analyzed. Subsequently, the calculated enthalpy was added to the enthalpy of the pure polyester substrate in order to obtain the total melting enthalpy ($∆H_{T}$). Furthermore, the crystallinity of yarns was calculated following Equation (1).

(1) $$\alpha \;(\%)=\frac{∆H_{T}}{117.6}\cdot 100$$

where 117.6 J/g is the enthalpy of a perfect polyester crystal. In all cases, the percentage of polyester in each blend was considered.

UPF determination

The UPF (UV protection factor) of the fabrics was measured on a Labsphere (North Sutton, NH, USA) UV Transmittance Analyzer (UPF) and evaluated with the specific program for UV-1000F fabrics, according to the AS/NZS 4399:1996 standard [34]. The spectral range of the instrument was set from 250 to 450 nm with a 10 mm viewing beam diameter. The reference of the solar spectrum used was the spectral irradiance according to CIE No. 85 measured in Albuquerque at noon on 3 July.

## 3. Results and Discussion

**Figure 1:**
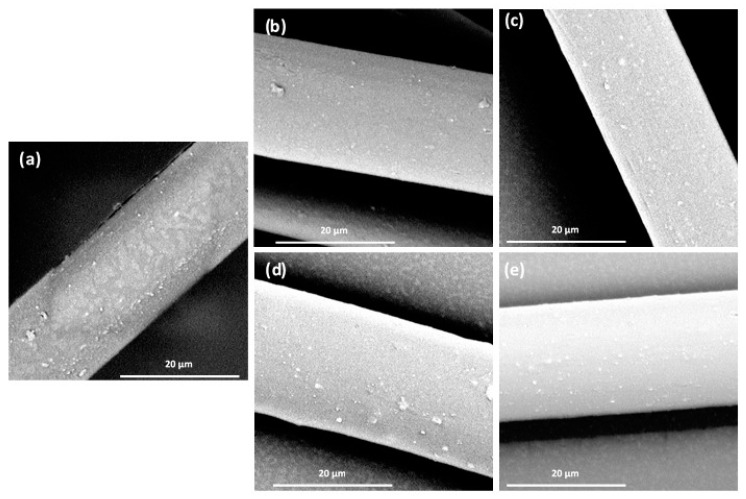
SEM images of (**a**) PET, (**b**) PET-RN, (**c**) PET-RM, (**d**) PET-AN, and (**e**) PET-AM.

Initially, micrographs of the drawn yarns were captured to determine whether any variations could be identified among the samples (Figure 1).

No noticeable variations were observed between the plain polyester and the polyester containing TiO_2_ particles. This suggests that the particles were evenly distributed within the fiber, indicating the effective role of the MAWMA dispersing agent. Conversely, the average molecular weight in number ($\bar{M_{n}}),\;\;in$ weight ($\bar{M_{w}}$), and the polydispersity ($\bar{M_{w}}$/$\bar{M_{n}}$) of the yarns were measured to assess any changes in the polymeric substrate (Table 4).

The results showed that the incorporation of ceramic particles caused a decrease in the molecular weight of the substrate. For the same manufacturing process, PET nanocomposites show a reduction in their intrinsic viscosity and, consequently, in their molecular weight compared to the pure polymer, which means that these materials are more sensitive to degradation as a result of heating, high shear rates and residence times during the extrusion process [35,36]. Comparing the TiO_2_-containing yarns, PET-RM, PET-AM and PET-AN exhibited very similar $\bar{M_{n}}$ values. In contrast, the PET-RN sample had the lowest values of $\bar{M_{n}}$ and $\bar{M_{w}}$. During the production of the composites, a decrease in viscosity was observed, leading to a necessary decrease in the concentration of rutile-type nanoparticles in the mixture. The polydispersity values for all samples were found to be 2.0, which is in accordance with the information available in the bibliography [35,37].

The thermal behavior of the samples was assessed using the Differential Scanning Calorimetry (DSC) technique. This analysis is crucial in establishing the effective temperature range for processing purposes. By examining the thermograms obtained from all samples, the temperature of crystallization (*T_c_*) and the enthalpy of crystallization (Δ*H_c_*) were determined using the methodology developed by Cayuela et al. [33] (Table 5).

Many important properties of polyesters depend on the nucleation process, in which the primary nuclei of the crystal are formed. This phenomenon can be homogeneous if the molecular movements of the polymer in the molten state cause the alignment of a sufficient number of segments of the chain to follow a more stable form; or heterogeneous if the nuclei are formed from the simple contact of the substance with the container or from insoluble microscopic particles randomly distributed in the material [38]. In the present case, TiO_2_ particles acted as heterogeneous crystallization nuclei, shifting the crystallization temperature towards higher values. In this context, the anatase form showed a higher crystallization temperature, which meant a greater nucleation power compared to the rutile form. Concretely, PET-AM was the sample with the highest value, with a crystallization temperature of 206.3 °C and a crystallization enthalpy of −54.7 ± 0.4 J/g.

The mechanical properties required for a fiber to have textile qualities are achieved through the drawing process. Hence, dynamometric tests were conducted on the yarns before and after this process (Table 6).

**Figure 2:**
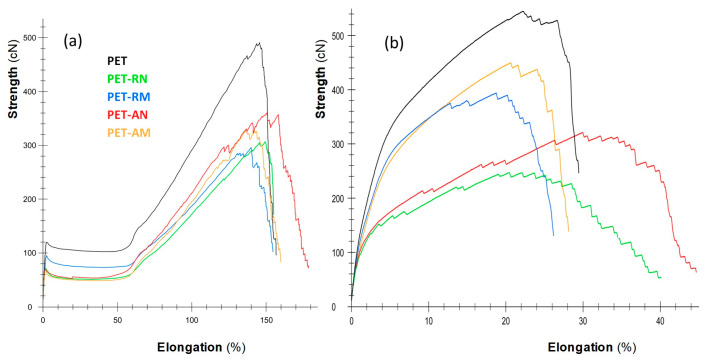
Stress–strain curves of (**a**) non-drawn and (**b**) drawn yarns.

In all instances, a reduction in the values of lineal density was observed after the drawing process. Nevertheless, no correlation could be established among the size, the polymorphism of the particles and the mentioned parameter, probably due to the effect of the dispersing agent, which provides a plasticizing effect to the macromolecular chains. In general, the tenacity and elongation slightly decreased after the incorporation of particles. A more comprehensive understanding of these changes was obtained by examining the stress–strain curves (Figure 2).

When compared to the rest, the yarn without particles exhibited a higher modulus and greater tensile strength. The inclusion of the particles decreased the resistance of the yarn, although the elongation was almost the same. Furthermore, it was observed that yarns with anatase TiO_2_ had better resistance properties than yarns with rutile particles. The yarns subjected to the drawing process decreased in resistance with respect to the non-drawn yarns. The decrease in magnitude was more significant when nano-sized particles were present rather than micro-sized particles regardless of the crystalline form. In addition, the presence of nanoparticles resulted in a higher elongation at break. This can be attributed to the smaller sizes of these particles, which caused a reduced obstacle effect compared to the microparticles.

In general, the tenacity of the yarns with particles was found to be lower compared to the tenacity of pure PET yarn. Previous investigations have indicated that when the quantity of ceramic particles, specifically TiO_2_, in Nylon yarns exceeds 0.33%, there is a decrease in the tensile strength. This reduction can be attributed to the presence of high particle concentrations, in this case ranging from 1.8 wt.% to 2 wt.%, which could lead to interactions and agglomerations. Consequently, the contact surface between the particles and the matrix is reduced. Furthermore, these particles can also serve as sites for stress concentration. As a result, when the material is subjected to tensile deformation, the larger particles induce higher stress concentrations in the matrix, leading to a decrease in impact energy. This ultimately causes the material to break earlier than expected [39,40,41].

The tenacity values of the yarns with microparticles were higher than those of yarns with nanoparticles, with these differences being more evident after drawing. This finding was correlated with the previously observed decrease in molecular weight values in the presence of particles. Despite the decrease in the mechanical properties, the tenacity values were still suitable for textile applications.

The yarns containing microparticles exhibited greater tenacity values in comparison to the yarns containing nanoparticles. The disparities between these two types of yarns became more apparent after undergoing the drawing process. This outcome can be attributed to the decrease in molecular weight values that were previously observed when particles were present. Despite the decline in mechanical properties, the tenacity values remained suitable for use in textile applications.

The sonic module technique enables the determination of the speed at which sound or a pulse passes longitudinally through a fiber. This is useful to determine the orientation of molecules since a higher speed corresponds to a greater orientation and value of the sonic module (Table 7).

The results indicated that regardless of the presence and type of particles, the non-drawn yarns exhibited similar orientation values. Nevertheless, the drawn yarns demonstrated two distinct behaviors: the original PET and the microparticle-containing yarns displayed similar orientation values, which were higher than those of the nano-sized counterparts. These findings can be linked to the values obtained from the dynamometric assay, particularly the greater elongation response observed in yarns containing nano-sized particles. The nanometric size of the particles potentially led to a higher degree of interlocking within the polymeric matrix, consequently hindering the orientation of molecules to a greater extent. Moreover, this result was consistent with the observed tenacity, indicating that yarns with microparticles exhibited higher tenacity compared to yarns with nanoparticles.

Regarding the crystallinity of non-drawn yarns (Table 7), notably, all the substrates examined showed no significant variations, except for the PET-AM sample, which displayed a higher level of crystallinity. It is worth noting that thermograms of undrawn fibers generally exhibited distinct thermal transitions. The first transition is characterized by a change in heat flux (heat capacity, c_p_) corresponding to the glass transition (Tg) region. This region is associated with the temperature at which the mobility of the polymer macromolecules begins in the amorphous zone of the fiber. This transition corresponds to the amorphous fraction in a triphasic model of the fiber. Subsequently, an exothermic peak is observed, indicating cold crystallization. This peak is related to the mesophase of the model, which represents the fraction of macromolecules that are ordered. By applying energy in the form of temperature, these macromolecules can crystallize due to their movement caused by thermal agitation. Finally, the melting endotherm observed in the non-drawn polyester thermograms indicates the melting of both the original crystallites and those formed during the cold crystallization transition.

**Figure 3:**
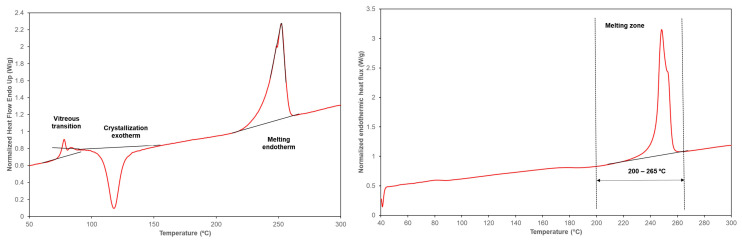
Thermograms corresponding to (**left**) thermal transitions of undrawn fibers and (**right**) drawn fibers.

The thermograms of the drawn samples did not display any distinct regions corresponding to the glass transition temperature (Tg) or cold crystallization (Figure 3). The process of drawing the fiber leads to the alignment of macromolecules along the fiber axis, thereby inducing a crystallization process. As a result, the intensity of thermal transitions associated with the amorphous portion of the polymer decreases. Additionally, this alignment of macromolecules in an extended state may facilitate the approximation of inter-macromolecular chains and the formation of bonds between groups. Consequently, the drawn yarns exhibit significantly higher crystallinity (five to eight times higher, depending on the case) compared to their precursors, in accordance with the increased orientation indicated by the sonic modulus results.

**Figure 4:**
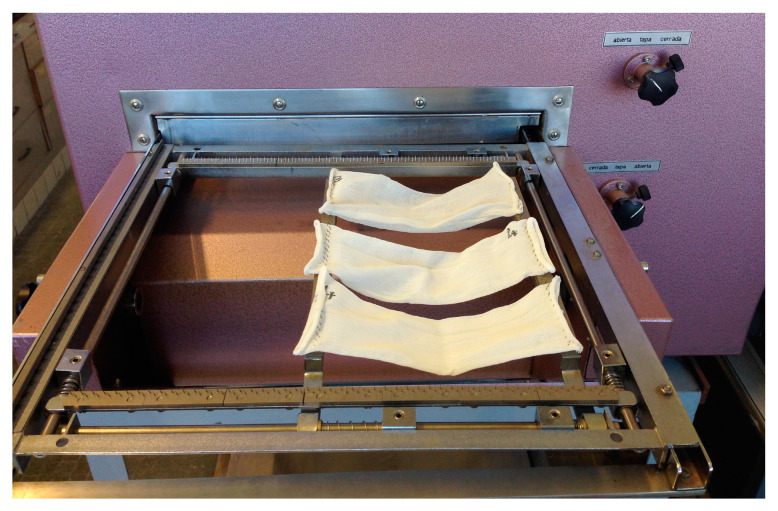
Knitted fabrics at the entrance of the stenter.

Once the production process was completed, the knitted fabrics underwent heatset (Figure 4). The evaluation of the impact of heatsetting on crystallinity (Table 7) indicated no significant variations among the different samples. This suggests that the thermomechanical treatments applied to the fibers, such as shrinkage and heatsetting, in order to confer textile properties to the TiO_2_-containing yarns, resulted in a final product with equivalent textile properties. Consequently, the inclusion of TiO_2_ particles during the spinning process altered the microstructure of the initial yarns, but these differences were eliminated during subsequent production processes, leading to the attainment of fabrics with comparable structures that can be dyed and finished under standard conditions for conventional polyester.

The UPF and UPF index of knitted fabric were determined, and the corresponding results are presented in Table 8.

**Figure 5:**
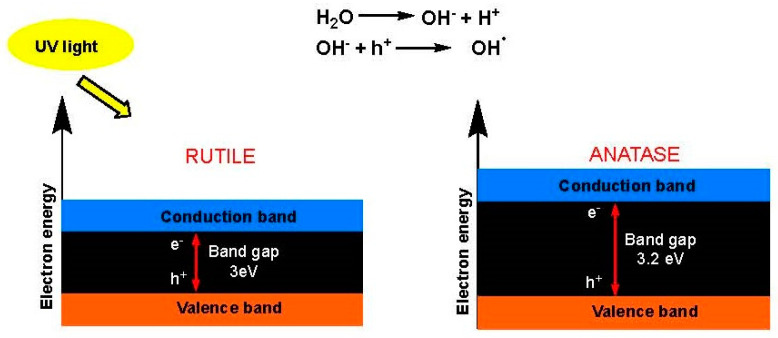
Band gaps of TiO_2_ in the crystalline forms of rutile (**left**) and anatase (**right**).

All fabrics with particles exhibited a significant improvement in the UPF value compared to the value of pure PET fabric. It was found that the fabric with PET-RM had the highest UPF value, suggesting that the rutile form had a greater protective effect than the anatase form. In terms of particle size, micro-sized particles had a greater protective effect than their nano-sized counterparts. The primary reason for the enhanced UV radiation protection provided by the rutile structure, in comparison to anatase, lies in the narrower band gap exhibited by rutile as opposed to anatase [25] and, as a result, rutile has a greater degree of electron–hole recombination. For this reason, the rutile phase is more suitable to be used as a UV blocker since the narrower the band, the better the behavior for blocking UV radiation (Figure 5).

**Figure 6:**
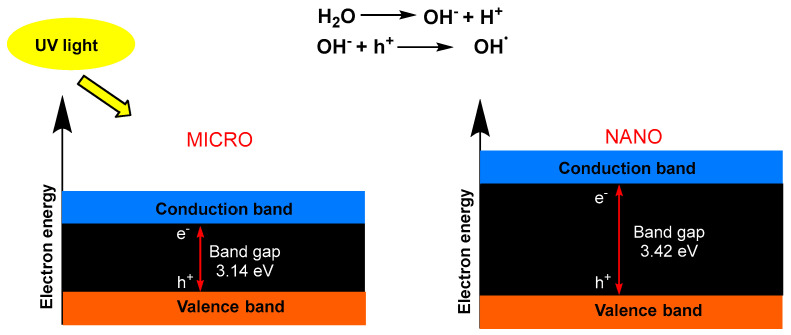
Band gaps of TiO_2_ particles in the size of (**left**) micrometers and (**right**) nanometers.

Regarding the size, in the same crystalline form, higher UPF values were observed in the micro-sized particles. This was related to the fact that, with larger particle sizes, the band energy decreases; thus, it excites the probability of electron–hole recombination to a higher degree, and therefore, the blocking of UV radiation is greater [42] (Figure 6).

## 4. Conclusions

In this manuscript, the inclusion and evaluation of the mechanical, thermal and UV blocking behavior of PET and PET-containing TiO_2_ with different sizes in rutile and anatase forms have been carried out. Firstly, micro- and nano-sized rutile and anatase TiO_2_ particles have been included in a PET matrix to produce multifilament yarns. MAWMA was found to be a suitable dispersing agent for these systems, achieving a good particle distribution. The molecular weight of the yarns was determined, and it was concluded that the inclusion of ceramic particles in PET caused a decrease in the molecular weight of the yarns and, as a consequence, a decrease in the tenacity of the yarns. In this way, the mechanical properties of the samples were affected by the presence of TiO_2_ particles; however, the yarns were still suitable for textile applications. The tenacity values of the yarns with microparticles were higher than those of the nano-sized homologues.

On the other hand, in the post-spinning drawing, the orientation of the macromolecules in the presence of nanoparticles was lower than that in the other substrates, as indicated by the lower value of the sonic modulus. It could be concluded that the nano-sized particles may hinder the alignment of the polymer chains. Regarding crystallization, a slight increase was observed in the drawn yarns containing particles, being higher in the PET-AN yarns, which act as nuclei of heterogeneous crystallization.

Furthermore, the influence of crystalline form and particle size on the UPF has been demonstrated. In all cases studied, the UPF values increased in the presence of TiO_2_ particles. The rutile crystalline form was found to be more effective in improving UV protection than the anatase crystalline form. Concomitantly, micro-sized particles were found to be more efficient than the nano-sized particles in terms of UV protection.

## Figures and Tables

**Table 1:** Characteristics of TiO_2_ particles.

	RN	RM	AN	AM
Commercial reference	MT-500HD Tayca Corporation	KRONOS 2360	AMT-600 Tayca Corporation	KRONOS 1071
Particle size (nm)	30	190	30	220
Crystalline form	Rutile	Rutile	Anatase	Anatase
Specific surface area (m^2^/g)	48	13–17	52	9–11
Composition (%)	TiO_2_: 85 Al_2_O_3_: 1–15 ZrO_2_: 1–10	TiO_2_ ≥ 92 Al_2_O_3_: 3–3.8 SiO_2_: 2.4–3 C: 0.18–0.2	TiO_2_: 80–98	TiO_2_ ≥ 96 Al_2_O_3_: 1–1.2 SiO_2_: 0.5–0.7 P_2_O_5_: 0.3–0.4 C: 0.15–0.25

**Table 2:** Characteristics of MAWMA dispersing agent.

Characteristics	Value
Appearance	Pale yellow pellets
Acid Index (mg KOH/g)	15–20
Saponification index (mg KOH/g)	140–160
Viscosity (mPa·s)	~20
Density (23 °C) (g/cm^3^)	~1.02

**Table 3:** Properties of manufactured POY PET yarns.

Sample	TiO_2_ (%)	Dispersing Agent (%)	Linear Density of Non-Drawn Yarn (tex)	Linear Density of Drawn Yarn (tex)
RN	1.8	1.8	26.9	17.6
RM	2	2	23.7	14.1
AN	2	2	27.4	18.3
AM	2	2	27.7	17.0
PET	--	--	28.9	15.8

**Table 4:** Values of $\bar{M_{n}}$, $\bar{M_{w}}$ and polydispersity of the yarns.

Sample	$\bar{M_{n}}$ (kg/mol)	$\bar{M_{w}}$ (kg/mol)	Polydispersity ($\bar{M_{w}}/\bar{M_{n}}$)
PET-RN	21.0	42.9	2.0
PET-RM	23.0	45.1	2.0
PET-AN	23.3	46.7	2.0
PET-AM	23.2	44.4	2.0
PET	27.6	51.5	2.0

**Table 5:** Temperature and enthalpy of crystallization from melting of mixtures.

Sample	*T_c_* (°C)	Δ*H_c_* (J/g)
PET-RN	202.8	−52.4 ± 0.2
PET-RM	205.3	−52.7 ± 1.2
PET-AN	206.3	−52.9 ± 0.4
PET-AM	206.3	−54.7 ± 0.4
PET	200.3	−48.4 ± 0.3

**Table 6:** Lineal density and tenacity values of non-drawn and drawn yarns.

Sample	Lineal Density (tex)		Tenacity (cN/dtex)		Elongation (%)	
	Non-Drawn	Drawn	Non-Drawn	Drawn	Non-Drawn	Drawn
PET-RN	26.9	17.6	1.14 ± 0.12	1.4 ± 0.2	150.9 ± 9.2	20.6 ± 3.9
PET-RM	23.7	14.1	1.25 ± 0.11	2.8 ± 0.2	140.1 ± 7.2	17.5 ± 2.4
PET-AN	27.4	18.3	1.32 ± 0.12	1.8 ± 0.3	148.7 ± 6.1	27.2 ± 4.7
PET-AM	27.7	17.0	1.18 ± 0.10	2.6 ± 0.2	140.3 ± 6.2	21.5 ± 2.1
PET	28.9	15.8	1.73 ± 0.11	3.5 ± 0.1	149.5 ± 8.2	27.6 ± 2.3

**Table 7:** Sonic modulus and crystallinity of studied yarns.

Sample	Sonic Modulus		Crystallinity (%)		
	Non-Drawn	Drawn	Non-Drawn	Drawn	Heatset Fabric
PET-RN	28.4 ± 0.2	86.2 ± 0.5	6.5	50.9	52.0
PET-RM	27.4 ± 1.5	106.6 ± 6.7	6.0	47.4	51.0
PET-AN	28.0 ± 2.0	91.0 ± 4.6	7.7	51.0	52.0
PET-AM	28.1 ± 0.8	106.7 ± 7.2	11.7	49.7	54.9
PET	26.1 ± 0.7	106.2 ± 6.1	8.0	47.1	53.5

**Table 8:** UPF values for the heatset fabrics.

Fabric	UPF	UPF Index
PET-RN	108	50+
PET-RM	165	50+
PET-AN	66	50+
PET-AM	105	50+
PET	33	30

## Data Availability

Data are contained within the article.
